# The Book World of Medicine and Science

**Published:** 1898-02-12

**Authors:** 


### THE BOOK WORLD OF MEDICINE AND SCIENCE.

SIR JAMES YOUNG SIMPSON AND CHLOROFORM. By H. LAING GORDON. Masters of Medicine Series. (London: T. Fisher Unwin. 1897. Price 3s. 6d.)

No one can say that this is not an interesting work. Perhaps one may say that it would not be altogether easy to write a life of Simpson which would not be interesting as a story of push, energy, and success. Nevertheless, we think it fails to some extent to give a proper presentment of its subject from the very fact that it is too obviously a panegyric. Like sugar to the pastrycook, after the first few pages praise falls flat. The life, however, is well told. How the young son of the Bathgate baker was marked out from his early days as likely to be famous, how he was set upon the path to greatness by his admiring family, was protected in his childhood, and guided in his youth with the one definite object constantly in view, and how he worked upwards and onwards till he obtained wealth, position, and world-wide fame, is all well described. The name of Simpson is known all the world over, and will always be remembered as the discoverer of the anæsthetic properties of chloroform. It must, however, be admitted that the time was ripe. The possibility of producing anæsthesia had already been demonstrated, but ether was hampered with many drawbacks. Something more handy was required, and every credit is due to Simpson for the pains he took and for the courage he displayed in his efforts to discover it. But so far as one can gather from the history given he does not seem to have set to work on any scientific plan or with any great depth of knowledge. He was convinced that something better than ether could be found, and he set to work to find it by inhaling "substance after substance." Appeal was made to chemists to provide all sorts of curious drugs, such as "their special knowledge might suggest," and in after-dinner séances night after night Simpson and his fellow "enthusiasts sat at the table and inhaled the particular substance under trial," but we find no record in the volume before us of the recognition of any principle on which the search was to be conducted. At last by good luck chloroform was hit upon. The discovery is described as a pure accident. One night, "having inhaled several substances, but without much effect, it occurred to Dr. Simpson to try a ponderous material which he had formerly set aside on a lumber table, and which on account of its great weight he had hitherto regarded as of no likelihood whatever; that happened to be a bottle of chloroform," and the discovery was made! This book has a good subject. It is a good tale well told, and shows how energy, self-confidence, and untiring industry will make their way in the world. It also gives many interesting details about its hero and his discoveries. But we think its author shows a too great readiness to fall down and worship.

ANNUAL REPORT OF THE MEDICAL OFFICER OF HEALTH OF THE ADMINISTRATIVE COUNTY OF LONDON, 1896. (London: Jas. Truscott and Son.)

This annual report is an elaborate and lengthy document full of facts and figures relating to the work of the medical department of the London County Council. The population of the county, which for sanitary purposes includes the City of London, estimated to the middle of 1896 was 4,443,311. The number of marriages in 1896 was 39,869, giving an annual rate of persons married of 18 per 1,000 living, which was higher than for some years previously. The births were 136,223 in number, giving an annual birth-rate of 30·2 per 1,000 living. The deaths numbered 81,963, giving an annual death-rate of 18·1 per cent.

The distribution of these deaths is interesting, although the deductions to be drawn from it are not so simple and straightforward as some people may be inclined to imagine. Taking the mortality of the whole of London as the standard, and representing it by the figure 1,000, Mr. Shirley Murphy has had the comparative mortality for the different districts worked out on the same basis, and thus is able to show by simple figures the death-rates of the various parts of the metropolis as compared with that of the whole.

The results are certainly striking. Thus while Hampstead, Lee, Lewisham, and Stoke Newington are represented by figures lying between 705 and 751, Holborn stands at 1,316, St. George, Southwark, at 1,373, St. George-in-the-East at 1,321, and St. Luke at 1,404. If we take the population as they stand, with all their inequalities as to age and sex, we have the startling fact that of a given number of persons picked haphazard out of the parish of St. Luke, more than twice as many will be dead before the year is out than will die out of the same number chosen in the same way in Hampstead, and nearly twice as many as in Stoke Newington. But even if we take into consideration all the corrections necessary for age and sex, we still find the same striking difference, an open district like Stoke Newington giving a mortality figure of 705, against one of 1,404 in central and closely-packed St. Luke.

And here we come across a curious thing which should make us hesitate much before jumping at conclusions from statistics. This parish of St. Luke, with the highest death-rate in London, has plenty of children, yet the infants do not die even at the average rate of London infants. So far, then, as the deaths of infants within a year of their birth can say anything about the sanitary condition of the homes, this low infantile death-rate would suggest that the heavy general mortality in St. Luke is due to economic rather than health conditions. Under the head of "Small-pox," details are given regarding various small outbreaks which took place in the course of the year. Commenting on the advantage of some age being fixed for the performance of revaccination, Mr. Murphy points out that as things stand at present the question whether a child shall be revaccinated is probably never seriously considered in many homes in the absence of any requirement as to the performance of revaccination. There is no period in the child's life when the parent has to come to a definite decision in the matter, and hence in many cases, from sheer apathy, revaccination is neglected. The number of parents who even think about the subject is probably small, the greater number having never considered the matter at all; but if it were required by the State that every child approaching the age of twelve should be revaccinated, even although the same provision were made for the conscientious objector as may be adopted in the case of primary vaccination, a vast number of children would be revaccinated whose parents now never think of having it done.

In the portion of the report dealing with puerperal fever, attention is drawn to the fact that a comparison of the fluctuations of the ratio of puerperal fever with those of erysipelas during the past six years shows a close correspondence between these two diseases, the prevalence of both of them rising and falling together year by year.

It is impossible in this review to go over more than a mere fraction of the ground covered by this most interesting report. Besides giving a record of the various diseases from which the inhabitants of the metropolis have suffered, a great deal of space is devoted to an account of the administrative work of the Health Department. Dairies, cowsheds, and milkshops, offensive trades, nuisances (a most elastic term), the housing of the working classes, the various schemes in progress for dealing with unhealthy areas, the regulation of houses let in lodgings, the supervision of common lodging-houses, the sanitary inspection of factories, workshops, and bakehouses, water supply, river pollution, and a host of other things come under the eye of the medical officer of health and those working under him, and no one can read this report and its appendices, and note the care that has been taken to obtain the fullest and most useful information from the statistics open to its authors, without recognising how great is the debt owed by London to the medical scientific adviser of its Council for the constant supervision exercised by him over all matters appertaining to the public health.

